# Correction to: Mindray 3-directional NMT Module (a new generation “Tri-axial” neuromuscular monitor) versus the Relaxometer mechanomyograph and versus the TOF-Watch SX acceleromyograph

**DOI:** 10.1007/s10877-019-00273-4

**Published:** 2019-02-26

**Authors:** Ashraf A. Dahaba, Ismet Suljevic, Zhao Yang Xiao, Kun Wang

**Affiliations:** 1 Department of Anaesthesiology and Intensive Care Medicine, Medical University of Graz, Auenbruggerplatz 29, 8036 Graz, Austria; 2 Department of Anaesthesiology, Sarajevo University Clinical Centre, Sarajevo, Bosnia and Herzegovina; 3 Department of Emergency Medicine, Dalian Medical University, Dalian, People’s Republic of China; 4 Laboratory of Pharmacometrics, Centre for Drug Clinical Research, Shanghai University of Traditional Chinese Medicine, Shanghai, 201203 People’s Republic of China

## Correction to: Journal of Clinical Monitoring and Computing 10.1007/s10877-018-0231-3

In the original publication of the article, the article note “Ashraf A. Dahaba and Zhao Yang Xiao equally contributed to the study and are both first authors.” was published incorrectly. The correct statement should read as “Ashraf A. Dahaba and Zhao Yang Xiao equally contributed to the study and are both first authors and are both co-corresponding authors.”

